# Clinical next generation sequencing of pediatric-type malignancies in adult patients identifies novel somatic aberrations

**DOI:** 10.18632/oncoscience.131

**Published:** 2015-02-20

**Authors:** Jorge Galvez Silva, Fernando F. Corrales-Medina, Ossama M. Maher, Nizar Tannir, Winston W. Huh, Michael E. Rytting, Vivek Subbiah

**Affiliations:** ^1^ Division of Pediatrics, The University of Texas MD Anderson Children's Cancer Hospital, Houston, TX; ^2^ Division of Pediatric Hematology-Oncology, Department of Pediatrics, University of Miami-Miller School of Medicine, Miami, FL; ^3^ Department of Genitourinary Medical Oncology, Division of Cancer Medicine, The University of Texas MD Anderson Cancer Center, Houston, TX; ^4^ Department of Investigational Cancer Therapeutics, Division of Cancer Medicine, The University of Texas MD Anderson Cancer Center, Houston, TX

**Keywords:** Next generation sequencing, Somatic Mutation, Solid Tumors

## Abstract

Pediatric malignancies in adults, in contrast to the same diseases in children are clinically more aggressive, resistant to chemotherapeutics, and carry a higher risk of relapse. Molecular profiling of tumor sample using next generation sequencing (NGS) has recently become clinically available. We report the results of targeted exome sequencing of six adult patients with pediatric-type malignancies : Wilms tumor(n=2), medulloblastoma(n=2), Ewing's sarcoma( n=1) and desmoplastic small round cell tumor (n=1) with a median age of 28.8 years. Detection of druggable somatic aberrations in tumors is feasible. However, identification of actionable target therapies in these rare adult patients with pediatric-type malignancies is challenging. Continuous efforts to establish a rare disease registry are warranted.

## INTRODUCTION

Pediatric oncologic diseases such as Ewing sarcoma (ES), Wilms tumor (WT), and medulloblastoma are infrequently found in adults. Pediatric malignancies in adults have proven to be more aggressive, resistant to chemotherapeutics, and to have a higher risk of relapse. Thus, they are very challenging to manage [1, 2]. NGS is an appealing available test that identifies oncogenic mutations, providing some insights in cancer biology and in some cases possible actionable targeted therapies.

We conducted a retrospective chart review of adult patients who were diagnosed as having pediatric- type malignancies and were referred to the Center for Targeted Therapy and/or the Division of Pediatrics at The University of Texas MD Anderson Cancer Center. Archived tumor samples with confirmed pathology were analyzed with use of Clinical Laboratory Improvement Amendments-Next-generation sequencing at Foundation Medicine, Cambridge, MA, USA.

## RESULTS

NGS profiling for six patients was available for review (Table 1). Median age at presentation was 28.8 years (23–38 years). Four of the six patients were male. The malignancies in all patients were solid tumors. Of the six analyzed samples, five were from the primary site; only one tumor was from a metastatic site in the liver of a patient with medulloblastoma. Pathology was confirmed via morphology and support with RT-PCR and immunohistochemistry for all of the samples. All patients were treated with chemotherapy and radiotherapy prior to tumor NGS analysis. No germline DNA sequence was available.

One patient with ES harbored *CDKN2A/B gene loss* and *BCL2L2* and *c17orf39 gene* amplifications. Of the two cases with WT, one showed *CTNNB1-*T257I which is located in the 3^rd^ armadillo repeat of beta-catenin gene, *IGF1R-*R595H which is a missense mutation in the alpha chain of the receptor, and *FAM123B-*R353* with *SPEN-*Q1122* that has never been reported before in genome data bases. The second patient harbored the *WT1 gene* mutation. The genetic aberrations in the two patients with a history of medulloblastoma were one patient with *BRCA1*-splice site 4987-1 G>A, which corresponds to the splice acceptor site near to C-terminus and a second patient with two different alterations in the *PTCH-1* gene, N97fs*43 and K163fs*6. One patient with desmoplastic small round cell tumor (DSRCT) harbored the novel *AURKB* and *MCL1* gene amplifications.

**Table 1:** Tumor Type Associated with Somatic Genetic Mutations

Diagnosis	Age	Tissue sample	Radiation	Initial chemotherapy	Genomic alteration
1. Bone Ewing sarcoma	23	Primary tumor	Yes	VDI	*CDKA2A/B* loss, *BCL2L2* amplification, c17orf39 amplification
2. Kidney Wilms tumor	36	Primary tumor	Yes	DD4A	*CTNNB1-*T257I, *IGF1R-*R595H, *FAM123B-*R353*, *SPEN*-Q1122
3. Kidney Wilms tumor	38	Primary tumor	Yes	DD4A	*WT1* mutation
4. Brain medulloblastoma	23	Metastasis/liver	Yes	Vincristine, carboplatin, TMZ	*BRCA*-splice site 4987-1G>A
5. Brain medulloblastoma	29	Primary tumor	Yes	Vincristine, CNNU, cisplatin	*PTCH*-1/N97fs*43 and K163fs*6
6. Soft tissue DSRCT	25	Primary tumor	Yes	VDC	*AURKB* amplification, *MCL1* amplification (BCL2 family)

DD4A-vincristine, doxorubicin, dactinomycin; CNNU-lomustine; VDC-vincristine, doxorubicin, cyclophosphamide; VDI-vincristine, doxorubicin, ifosfamide; TMZ-temozolomide.

Clinical trials and possible off label FDA-approved drugs for the entire potential proposed target therapies were researched (Table 2). For patients with ES with CDKN2A/B gene alterations possible targets for the molecules CDK4 and CDK6 were found, but no targets are available for gene loss of function. No possible targets were found for the BCL2L2 or c17orf39 amplifications. For the patient with WT who harbored CTNNB1 no therapies were found. IGF1R, possible targets include small molecules inhibitors in early clinical studies[3-5], whereas for the patient with WT-1 mutation, WT-1 pathway peptides are still under basic research and early clinical studies[6]. In the case of the medulloblastoma, BRCA1 mutations may be targeted with DNA damaging drugs such as platinum and PARP inhibitors that are currently in clinical trials for brain tumors[7-9]; furthermore, the PTCH-1 aberration seen in medulloblastoma could be targeted with SMO/SHH inhibitors such as vismodegib[10]. The DSRCT tumor harbored the AURKB and MCL1 gene amplifications with no approved therapies, nonetheless, there are few clinical trials targeting Aurora kinases and CDK inhibitors[11-13].

**Table 2:** Potential Target Therapies

Disease	Gene affected	Possible Targets Pathways	Possible therapies
Bone Ewing Sarcoma	CDKA2A/B mutation/loss BCL2L2 and c17orf39 amplification	CDK4, CDK6 Bcl-w	CDK4/6 inhibitors ^#^* Navitoclax*
Wilms Tumor	CTNNB1 IGF1R, FAM123B, SPEN Q1122	Beta-catenin, Wnt IGFR, EGF1R, MTOR No targets	PRI-724*, OMP-54F28*, AMG 479*, Everolimus, Temsirolimus, Sirolimus, Panitumumab
Wilms Tumor	WT1 Mutations	WT1	WT1-peptide–based immunotherapy, HLA-A*2404-restricted, 9-mer WT1 peptide
Medulloblastoma	BRCA	PARP	PARP inhibitors (rucaparib)*
Medulloblastoma	PTCH-1	SMO (SHH)	Vismodegib (GDC-0449)
Desmoplastic small round cell tumor	AURKB MCL1 **ARID1A, **RUNX1	Aurora kinase (chromosomal passenger complex)CSF1R, FLT3 PDGFRB, VEGFR-1-3; CDK1-4, 7, 9.	AMG 900* ASLAN002* Sorafenib, BAY 1000394*

* No US Federal Drug Administration (FDA) approval.

** Variance of unknown significance detected in the tumor sample.

# Unknown value in loss of function mutations.

## DISCUSSION

The recent advances in genomics have proved to be linked to prognosis and response to therapy, for instance BRAF inhibitors have changed the landscape of *BRAF* V600 E mutant melanomas[14, 15], and ALK inhibitors have dramatically changed the outcome of *EML4-ALK* mutant lung cancer patients[16]. NGS is a novel available technology that can provide valuable information leading to more accurate diagnosis, improved classification, and new biologic-based treatments. NGS could help in elucidating if the genetics of pediatric tumors may differ from that of adult tumors, even if the tumors for both groups are categorized as the same entities. This can be explained because many pediatric malignancies, when found in adult patients, may carry novel and/or more complex somatic mutations. For example, our patient with ES harbored *CDKA2A/B* loss, *BCL2L2* and *c17orf39* amplifications, with the latter amplifications never having been reported previously in patients with ES[17].

*CDKN2A/B* loss has appeared as an emergent mutation in ES that can be seen in 5%-12% of primary tumors and in up to 33%-50% of cell lines[18, 19]. Although Brownhill et al. did not show prognostic relevance in homozygous loss or single deletion of *CDKN2A*, other studies of aggressive sarcomas have shown an association between genomic alterations and disease progression [1, 2, 20]. In a pre-clinical model loss of CDKN2A expression correlated with sensitivity to CDK4/6 inhibitors[21]. However, clinical data is lacking[21].

*BCL2L2* amplification has never been found in ES. However, it has been associated with lower long-term survival in osteosarcoma [22]. *C17orf39* (GID4) amplification seen in our patient is another novel mutation for ES. This genomic event lies in the chromosome 17p11 frequently amplified in osteosarcoma and occasionally in gliomas [23, 24]. Currently, there are no targeted therapies available to address these aforementioned amplifications.

Our patient with WT harbored four alterations: *CTNNB1-*T257I, *IGF1R*-R595H*, FAM123B-*R353*, and *SPEN-*Q1122*. *CTNNB1* encodes for a protein named, beta-catenin, found in 15%-19% of patients with WT [25]. Some mutations in this gene such as T41A have been associated with significantly lower survival and resistance to chemotherapy in WT, however the biology effect in T257I is unknown [26].

*IGF1R*-R595H is a missense mutation in which the effect of the protein alteration is unknown. It has never been associated with WT. However, amplification of the tyrosine kinase has been observed in 10% of WT and has been associated with poor prognosis and relapse [27].

Somatic mutations in *FAM123* is rare in cancers genome databases, nonetheless is observed in 5-30% WT[28-30]. Overexpression of *SPEN*-Polyvalent transcriptional co-repressor have been suggested as an enhancer of the *Wnt* pathway, which has been also reported in colon and ovarian cancers.[28, 29, 31, 32] Currently, no targeted therapies are available.

Our patient with medulloblastoma was found to have *BRCA1*-splice site 4987-1-G>A that may lead to production of a truncated protein that prevents the *BRCT* domain from binding to several tumor suppressor proteins [33]. The *BRCA* mutation may be sensitive to DNA-damaging drugs such as platinum and PARP inhibitors [7]. Our second patient with medulloblastoma showed the *PTCH-1-* N97fs*43 and K163fs*6 mutations. Mutations in this gene have been found in 15% of medulloblastoma in genome databases. *PTCH* encodes for the *Ptc1* protein, a component of the hedgehog pathway. This pathway has been targeted with small molecules such as vismodegib, an inhibitor of the smoothened protein and a member of the hedgehog-signaling pathway. Rudin at el. reported tumor regression in 2009 after 3 months of therapy in patients with medulloblastoma [10].

Finally, our patient with DSRCT showed amplification in *AURKB* and *MCL1* genes. AURKB had never been associated with DSRCT. Decreased aurora kinase protein expression has been linked with poor response to chemotherapy in ovarian cancer [34].

MCL1 encodes for the protein *Mcl-1*, which is a member of the *Bcl-2* family. Amplification has been found in up to 10% of all tumors studied. It is more frequently found in aggressive tumors such as breast cancer and lung cancer [35].

NGS technology has helped identify additional genomics, epigenetics, and molecular aberrations that need more profound studies and analyses. Studies have shown that some alterations can be linked to prognosis in many tumors. Our understanding of the aggressive characteristics of pediatric malignancies in adults still has many gaps. This represents a challenge for physicians who seek to develop more individualized treatment strategies, as well as potential targets and clinical trials that will prove the safety and efficacy. Also, it would be of interest to analyze tumor samples pre-treatment as well as DNA germline sequence for each of the patients.

## CONCLUSION

Identification of somatic aberrations in adult patients with pediatric-type malignancies with use of CLIA-certified clinical NGS is feasible. Moreover, finding targeted therapies is complicated. Establishing a rare disease registry is warranted. In addition, further larger analyses of these types of patients such as The Cancer Genome Atlas along with clinical correlation are needed.

## PATIENTS AND METHODS

A retrospective electronic medical record review of adult patients with advanced metastatic pediatric-type malignancies was conducted. These charts were derived from patients who were referred to the Department of Investigational Cancer Therapeutics (Phase I Clinical Trials Program) and/or Division of Pediatrics. Tissue samples were based on archival samples from primary malignancy specimen or a biopsy from metastatic site. The University of Texas MD Anderson Cancer Center Institutional Review Board approval was obtained. All patients provided written informed consent for participation and for the chemotherapy or targeted therapy they received.

### Specimen analysis

Clinical targeted next-generation sequencing (NGS) analysis were performed by Foundation Medicine (Boston, MA) to identify genomic alterations within targeted 186 cancer-related genes.

### Statistical analysis

There is no formal hypothesis testing in this retrospective study. This is mainly a descriptive case series and we used descriptive statistics to report the findings.
